# Current Awareness on Comparative and Functional Genomics

**DOI:** 10.1002/cfg.120

**Published:** 2002-10


**Comparative and Functional Genomics**
*Comp Funct Genom* 2002; **3:** 461–468
Published online in Wiley InterScience (www.interscience.wiley.com). **DOI** 10.1002/cfg.120

### Current awareness on comparative and functional genomics

### 1 Reviews & symposia

2002. Special issue: Renal development and disease: From gene screening to functional genomics. *Exp Nephrol* **10:** (2)

2002. Special issue: XXXXIVth Annual Thomas I Petty Aspen Lung Conference: Pulmonary, Genetics, Genomics and Gene Therapy. *Chest* **121:** (3 Suppl)

Aigaki T, Ohsako T, Toba G, Seong KH, Matsuo T. 2001. Tokyo Metropolitan Univ, Dept Biol Sci, 1-1 Minami Osawa, Hachioji, Tokyo 192 0397, Japan. The gene search system: Its application to functional genomics in *Drosophila melanogaster*. *J Neurogenet* **15:** (3-4) 169.

Albers SV, Driessen AJM*. 2002. *Univ Groningen, Dept Microbiol, Kerklaan 30, NL-9751 NN Haren, The Netherlands. Signal peptides of secreted proteins of the archaeon *Sulfolobus solfataricus*: A genomic survey (Mini-Review). *Arch Microbiol* **177:** (3) 209.

Ball KD, Trevors JT*. 2002. *Univ Guelph, Dept Environ Biol, Guelph, Ontario, Canada N1G 2W1. Bacterial genomics: The use of DNA microarrays and bacterial artificial chromosomes. *J Microbiol Methods* **49:** (3) 275.

Bandara LR, Kennedy S. 2002. Oxford Glycosci UK, Forum, 86 Milton Pk, Abingdon OX14 4RY, England. Toxicoproteomics: A new preclinical tool (Review). *Drug Discov Today* **7:** (7) 411.

Chakravarti DN, Chakravarti B, Moutsatsos I. 2002. Wyeth Lederle Vaccines, Biotechnol Discovery Res Grp, 211 Bailey Rd, West Henrietta, NY 14586, USA. Informatic tools for proteome profiling (Review). *Biotechniques* (Suppl) 4.

Chance MR, Bresnick AR, Burley SK, Jiang JS, Lima CD, Sali A, Almo SC, Bonanno JB, Buglino JA, Boulton S *et al.* 2002. Albert Einstein Coll Med, Dept Biochem, 1300 Morris Pk Ave, Bronx, NY 10461, USA. Structural genomics: A pipeline for providing structures for the biologist. *Protein Sci* **11:** (4) 723.

Dierick JF, Dieu M, Remacle J, Raes M, Roepstorff P, Toussaint O*. 2002. *Fac Univ Notre Dame Paix, Lab Biochim & Biol Cellulaire, rue Bruxelles 61, BE-5000 Namur, Belgium. Proteomics in experimental gerontology. *Exp Gerontol* **37:** (5) 721.

Fielden MR, Matthews JB, Fertuck KC, Halgren RG, Zacharewski TR*. 2002. *Michigan State Univ, Dept Biochem & Mol Biol, Natl Food Safety & Toxicol Ctr, East Lansing, Mi 48824, USA. *In silico* approaches to mechanistic and predictive toxicology: An introduction to bioinformatics for toxicologists. *Crit Rev Toxicol* **32:** (2) 67.

Grant EP, Pickard MD, Briskin MJ, Gutierrez-Ramos JC. 2002. Millennium Pharmaceut, 75 Sidney St, Cambridge, Ma 02139, USA. Gene expression profiles: Creating new perspectives in arthritis research - Review. *Arthritis Rheum* **46:** (4) 874.

Graves PR, Haystead TAJ*. 2002. *Duke Univ, Dept Pharmacol & Canc Biol, Research Dr, Durham, NC 27710, USA. Molecular biologist's guide to proteomics (Review). *Microbiol Mol Biol Rev* **66:** (1) 39.

Hanash SM, Madoz-Gurpide J, Misek DE. 2002. Univ Michigan, Dept Pediat, 1150 West Med Ctr, Ann Arbor, Mi 48109, USA. Identification of novel targets for cancer therapy using expression proteomics. *Leukemia* **16:** (4) 478.

Heal JR, Roberts GW, Raynes JG, Bhakoo A, Miller AD*. 2002. *Univ London, Imperial Coll Sci Technol & Med, Genet Therapies Ctr, London SW7 2AZ, England. Specific interactions between sense and complementary peptides: The basis for the proteomic (Review). *Chembiochem* **3:** (2-3) 137.

Issaq HJ, Veenstra TD, Conrads TP, Felschow D. 2002. NIH/NCI, SAIC Frederick Inc, POB B, Bldg 469, Room 155, Frederick, Md 21702, USA. The SELDI-TOF MS approach to proteomics: Protein profiling and biomarker identification. *Biochem Biophys Res Commun* **292:** (3) 587.

Lacroix M, Zammatteo N, Remacle J, Leclercq G. 2002. VUB, Lab JC Heuson Cancerol Mammaire, 127 Blvd Waterloo, BE-1000 Brussels, Belgium. A low-density DNA microarray for analysis of markers in breast cancer (Review). *Int J Biol Markers* **17:** (1) 5.

Liebler DC. 2002. Univ Arizona, SW Environ Hlth Sci Ctr, 1703 East Mabel St, Tucson, Az 85721, USA. Proteomic approaches to characterize protein modifications: New tools to study the effect of environmental exposures. *Environ Health Perspect* **110:** (Suppl 1) 3.

Moldovan L, Moldovan NI*. 2002. *Heart & Lung Res Inst, 473 West 12th Ave, Columbus, Oh 43210, USA. Trends in genomic analysis of the cardiovascular system. *Arch Pathol Lab Med* **126:** (3) 310.

Moran NA. 2002. Univ Arizona, Dept Ecol & Evolutionary Biol, Tucson, Az 85721, USA. Microbial minimalism: Genome reduction in bacterial pathogens (Minireview). *Cell* **108:** (5) 583.

Nassif X. 2002. Univ Paris 05, Lab Microbiol, INSERM U411, 156 rue Vaugirard, FR-75730 Paris 15, France. Genomics of *Neisseria meningitidis*. *Int J Med Microbiol* **291:** (6-7) 419.

Palmer GH. 2002. Washington State Univ, Dept Vet Microbiol & Pathol, Pullman, Wa 99164, USA. The highest priority: What microbial genomes are telling us about immunity. *Vet Immunol Immunopathol* **85:** (1-2) 1.

Pirrung MC. 2002. Duke Univ, Dept Chem, Levine Sci Res Ctr, Durham, NC 27708, USA. How to make a DNA chip (Review). *Angew Chem Int Ed* **41:** (8) 1277.

Ramaswamy S, Golub TR*. 2002. *Harvard Univ, Dana Farber Canc Inst, 44 Binney St, Boston, Ma 02115, USA. DNA microarrays in clinical oncology. *J Clin Oncol* **20:** (7) 1932.

Reynolds MA. 2002. Incyte Genomics, 6519 Dumbarton Circle, Fremont, Ca 94555, USA. GEM™ microarrays and drug discovery. *J Ind Microbiol Biotechnol* **28:** (3) 180.

Robinson WH, Steinman L, Utz PJ. 2002. Stanford Univ, Beckman Ctr, Stanford, Ca 94305, USA. Proteomics technologies for the study of autoimmune disease (Review). *Arthritis Rheum* **46:** (4) 885.

Staudt LM. 2002. NCI, Ctr Canc Res, 9000 Rockville Pike, Bethesda, Md 20892, USA. Gene expression profiling of lymphoid malignancies (Review). *Annu Rev Med* **53:** 303.

Templin MF, Stoll D, Schrenk M, Traub PC, Vohringer CF, Joos TO. 2002. Univ Tubingen, NMI Nat & Med Sci Inst, Markwiesenstr 55, DE-72770 Reutlingen, Germany. Protein microarray technology (Review). *Trends Biotechnol* **20:** (4) 160.

Toda M, Ono SJ*. 2002. *UCL, Dept Ocular Immunol, 11-43 Bath St, London EC1V 9EL, England. Genomics and proteomics of allergic disease (Review). *Immunology* **106:** (1) 1.

Vreeland WN, Barron AE*. 2002. *Northwestern Univ, Dept Chem Engn, 2145 Sheridan Rd, Evanston, Il 60208, USA. Functional materials for microscale genomic and proteomic analyses. *Curr Opin Biotechnol* **13:** (2) 87.

Vuilleumier S, Pagni M. 2002. ETH Zurich, Inst Mikrobiol, Schmelzbergstr 7, CH-8092 Zurich, Switzerland. The elusive roles of bacterial glutathione S-transferases: New lessons from genomes (Mini-Review). *Appl Microbiol Biotechnol* **58:** (2) 138.

Weckwerth W, Fiehn O*. 2002. *Max Planck Inst, Dept Lothar Willmitzer, DE-14424 Potsdam, Germany. Can we discover novel pathways using metabolomic analysis? *Curr Opin Biotechnol* **13:** (2) 156.

Witzmann FA, Li JY. 2002. Indiana Univ, Dept Cellular & Integrat Physiol, 635 Barnhill Dr, Indianapolis, In 46202, USA. Cutting-edge

In order to keep subscribers up-to-date with the latest developments in their field, this current awareness service is provided by John Wiley & Sons and contains newly-published material on comparative and functional genomics. Each bibliography is divided into 16 sections. 1 Reviews & symposia; 2 General; 3 Large-scale sequencing and mapping; 4 Evolutionary genomics; 5 Comparative genomics; 6 Pathways, gene families and regulons; 7 Pharmacogenomics; 8 EST, cDNA and other clone resources; 9 Functional genomics; 10 Transcriptomics; 11 Proteomics; 12 Protein structural genomics; 13 Metabolomics; 14 Genomic approaches to development; 15 Technological advances; 16 Bioinformatics. Within each section, articles are listed in alphabetical order with respect to author. If, in the preceding period, no publications are located relevant to any one of these headings, that section will be omitted.

Copyright © 2002 John Wiley & Sons, Ltd.

technology - II. Proteomics: Core technologies and applications in physiology. *Am J Physiol* **282:** (5) G735.

### 3 Large-scale sequencing and mapping

Bentley SD, Chater KF, Cerdeno-Tarraga AM, Challis GL, Thomson NR, James KD, Harris DE, Quail MA, Kieser H, Harper D *et al.* 2002. Wellcome Trust Sanger Inst, Wellcome Trust Genome Campus, Cambridge CB10 1SA, England. Complete genome sequence of the model actinomycete *Streptomyces coelicolor* A3(2). *Nature* **417:** (6885) 141.

Chen M, Presting G, Barbazuk WB, Goicoechea JL, Blackmon B, Fang G, Kim H, Frisch D, Yu Y, Sun S *et al.* 2002. c/o Wing RA, Clemson Univ, Genomics Inst, 100 Jordan Hall, Clemson, SC 29634, USA. An integrated physical and genetic map of the rice genome. *Plant Cell* **14:** (3) 537.

Galagan JE, Nusbaum C, Roy A, Endrizzi MG, Macdonald P, FitzHugh W, Calvo S, Engels R, Smirnov S, Atnoor D *et al.* 2002. c/o Birren B, Whitehead Inst Ctr Genome Res, Cambridge, Ma 02141, USA. The genome of *M. acetivorans* reveals extensive metabolic and physiological diversity. *Genome Res* **12:** (4) 532.

Goff SA, Ricke D, Lan TH, Presting G, Wang R, Dunn M, Glazebrook J, Sessions A, Oeller P, Varma H *et al.* 2002. Syngenta, Torrey Mesa Res Inst, 3115 Merryfield Row, San Diego, Ca 92121, USA. A draft sequence of the rice genome (*Oryza sativa* L. ssp *japonica*). *Science* **296:** (5565) 92.

Kapatral V, Anderson I, Ivanova N, Reznik G, Los T, Lykidis A, Bhattacharyya A, Bartman A, Gardner W, Grechkin G *et al.* 2002. Integrated Genomics, 2201 West Campbell Dr Pk, Chicago, Il 60612, USA. Genome sequence and analysis of the oral bacterium *Fusobacterium nucleatum* strain ATCC 25586. *J Bacteriol* **184:** (7) 2005.

Slesarev AI, Mezhevaya KV, Makarova KS, Polushin NN, Shcherbinina OV, Shakhova VV, Belova GI, Aravind L, Natale DA, Rogozin IB *et al.* 2002. Fidel Syst, Gaithersburg, Md 20879, USA. The complete genome of hyperthermophile *Methanopyrus kandleri* AV19 and monophyly of archaeal methanogens. *Proc Natl Acad Sci U S A* **99:** (7) 4644.

Stone RT, Grosse WM, Casas E, Smith TPL, Keele JW, Bennett GL. 2002. USDA/ARS, US Meat Anim Res Ctr, POB 166, Clay Ctr, Ne 68933, USA. Use of bovine EST data and human genomic sequences to map 100 gene-specific bovine markers. *Mamm Genome* **13:** (4) 211.

Tauch A, Homann I, Mormann S, Ruberg S, Billault A, Bathe B, Brand S, Brockmann-Gretza O, Ruckert C, Schischka N *et al.* 2002. Univ Bielefeld, Zentrum Genomforsch, Universitatsstr 25, DE-33615 Bielefeld, Germany. Strategy to sequence the genome of *Corynebacterium glutamicum* ATCC 13032: Use of a cosmid and a bacterial artificial chromosome library. *J Biotechnol* **95:** (1) 25.

Wallace CA, Ali S, Glazier AM, Norsworthy PJ, Carlos DC, Scott J, Freeman TC, Stanton LW, Kwitek AE, Aitman TJ. 2002. Hammersmith Hosp, Physiol Genomics & Med Grp, Du Cane Rd, London W12 0NN, England. Radiation hybrid mapping of 70 rat genes from a data set of differentially expressed genes. *Mamm Genome* **13:** (4) 194.

West L, Yang D, Stephens C*. 2002. *Santa Clara Univ, Dept Biol, 500 El Camino Real, Santa Clara, Ca 95053, USA. Use of the *Caulobacter crescentus* genome sequence to develop a method for systematic genetic mapping. *J Bacteriol* **184:** (8) 2155.

Yu J, Hu S, Wang J, Wong GK, Li S, Liu B, Deng Y, Dai L, Zhou Y, Zhang X *et al.* 2002. c/o Yuan LP, Chinese Acad Sci, Inst Genet, CN-100101 Beijing, Peoples Rep China. A draft sequence of the rice genome (*Oryza sativa* L. ssp *indica*). *Science* **296:** (5565) 79.

### 4 Evolutionary genomics

Bansal AK, Meyer TE*. 2002. *Univ Arizona, Dept Biochem, Tucson, Az 85721, USA. Evolutionary analysis by whole-genome comparisons. *J Bacteriol* **184:** (8) 2260.

Clarke GDP, Beiko RG, Ragan MA*, Charlebois RL. 2002. *Univ Queensland, Inst Mol Biosci, Brisbane, Qld 4072, Australia. Inferring genome trees by using a filter to eliminate phylogenetically discordant sequences and a distance matrix based on mean normalized BLASTP scores. *J Bacteriol* **184:** (8) 2072.

De las Rivas J, Lozano JJ, Ortiz AR*. 2002. *NYU, Mt Sinai Sch Med, Dept Physiol & Biophys, New York, NY 10029, USA. Comparative analysis of chloroplast genomes: Functional annotation, genome-based phylogeny, and deduced evolutionary patterns. *Genome Res* **12:** (4) 567.

Feuillet C, Keller B. 2002. Univ Zurich, Inst Plant Biol, Zollikerstr 107, CH-8008 Zurich, Switzerland. Comparative genomics in the grass family: Molecular characterization of grass genome structure and evolution. *Ann Bot* **89:** (1) 3.

Stuart GW, Moffett K, Leader JJ. 2002. Indiana State Univ, Dept Life Sci, Terre Haute, In 47809, USA. A comprehensive vertebrate phylogeny using vector representations of protein sequences from whole genomes. *Mol Biol Evol* **19:** (4) 554.

### 5 Comparative genomics

Anantharaman V, Koonin EV*, Aravind L. 2002. *NIH/NLM, NCBI, 8600 Rockville Pike, Bethesda, Md 20894, USA. Comparative genomics and evolution of proteins involved in RNA metabolism. *Nucleic Acids Res* **30:** (7) 1427.

Bannantine JP, Baechler E, Zhang Q, Li LL, Kapur V. 2002. USDA/ARS, Natl Anim Dis Ctr, 2300 Nth Dayton Ave, Ames, Ia 50010, USA. Genome scale comparison of *Mycobacterium avium* subsp *paratuberculosis* with *Mycobacterium avium* subsp *avium* reveals potential diagnostic sequences. *J Clin Microbiol* **40:** (4) 1303.

Gomez-Fabre PM, Helou K, Stahl F*. 2002. *Univ Gothenburg, Dept Cell & Mol Biol Genet, SE-40530 Gothenburg, Sweden. Predictions based on the rat-mouse comparative map provide mapping information on over 6000 new rat genes. *Mamm Genome* **13:** (4) 189.

Lagerstrom-Fermer M, Larhammar D, Johnsen E, Landegren U. 2002. Univ Uppsala Hosp, Dept Med Sci, SE-75185 Uppsala, Sweden. Comparative genomics by capture PCR. *Genomics* **79:** (3) 432.

Van Buuren ML, Salvi S, Morgante M, Serhani B, Tuberosa R*. 2002. *Univ Bologna, Dipt Agroenvironm Sci & Technol, via Filippo Re 6-8, IT-40126 Bologna, Italy. Comparative genomic mapping between a 754 kb region flanking DREB1A in *Arabidopsis thaliana* and maize. *Plant Mol Biol* **48:** (5) 741.

### 6 Pathways, gene families and regulons

Cao M, Kobel PA, Morshedi MM, Wu MFW, Paddon C, Helmann JD*. 2002. *Cornell Univ, Dept Microbiol, Ithaca, NY 14853, USA. Defining the *Bacillus subtilis* $\sigma^W$ regulon: A comparative analysis of promoter consensus search, run-off transcription/macroarray analysis (ROMA), and transcriptional profiling approaches. *J Mol Biol* **316:** (3) 443.

### 7 Pharmacogenomics

Al Moustafa AE, Alaoui-Jamali MA, Batist G, Hernandez-Perez M, Serruya C, Alpert L, Black MJ, Sladek R, Foulkes WD. 2002. Sir Mortimer B Davis Jewish Hosp, Dept Med, Montreal, Quebec, Canada H3T 1E2. Identification of genes associated with head and neck carcinogenesis by cDNA microarray comparison between matched primary normal epithelial and squamous carcinoma cells. *Oncogene* **21:** (17) 2634.

Asakura M, Kitakaze M*, Sakata Y, Asanuma H, Sanada S, Kim J, Ogida H, Liao YL, Node K, Takashima S *et al.* 2002. *Osaka Univ, Dept Internal Med & Therapeut, 2-2 Yamadaoka, Suita, Osaka 565 0871, Japan. Adenosine-induced cardiac gene expression of ischemic murine hearts revealed by cDNA array hybridization. *Circ J* **66:** (1) 93.

Ball G, Mian S, Holding F, Allibone RO, Lowe J, Ali S, Li G, McCardle S, Ellis IO, Creaser C, Rees RC. 2002. Nottingham Trent Univ, Dept Life Sci, Clifton Lane, Nottingham NG11 8NS, England. An integrated approach utilizing artificial neural networks and SELDI mass spectrometry for the classification of human tumours and rapid identification of potential biomarkers. *Bioinformatics* **18:** (3) 395.

Bashyam MD. 2002. Ctr DNA Fingerprinting & Diagnost, Mol Oncol, IN-500076 Hyderabad, India. Understanding cancer metastasis: An urgent need for using differential gene expression analysis. *Cancer* **94:** (6) 1821.

Benson M, Svensson PA, Carlsson B, Jernas M, Reinholdt J, Cardell

Copyright © 2002 John Wiley & Sons, Ltd.

LO, Carlsson L. 2002. Malmo Univ Hosp, Dept Otorhinolaryngol, Allergy Lab, SE-20502 Malmo, Sweden. DNA microarrays to study gene expression in allergic airways. *Clin Exp Allergy* **32:** (2) 301.

Bernard FX, Pedretti N, Rosdy M, Deguercy A. 2002. BIOalternatives, 1 bis rue Plantes, FR-86160 Gencay, France. Comparison of gene expression profiles in human keratinocyte mono-layer cultures, reconstituted epidermis and normal human skin: Transcriptional effects of retinoid treatments in reconstituted human epidermis. *Exp Dermatol* **11:** (1) 59.

Clark J, Edwards S, John M, Flohr P, Gordon T, Maillard K, Giddings I, Brown C, Bagherzadeh A, Campbell C *et al*. 2002. c/o Cooper CS, Inst Canc Res, Haddow Labs, 15 Cotswold Rd, Sutton SM2 5NG, England. Identification of amplified and expressed genes in breast cancer by comparative hybridization onto microarrays of randomly selected cDNA clones. *Gene Chromosomes Cancer* **34:** (1) 104.

Curto EV, Lambert GW, Davis RL, Wilborn TW, Dooley TP. 2002. IntegriDerm Inc, 2130 Mem Pkwy SW, Huntsville, Al 35801, USA. Biomarkers of human skin cells identified using DermArray DNA arrays and new bioinformatics methods. *Biochem Biophys Res Commun* **291:** (4) 1052.

Dahse R, Berndt A, Kosmehl H. 2002. Klinikum FSU Jena, Inst Humangenet & Anthropol, Kollegiengasse 10, DE-07740 Jena, Germany. Total RNA-based target design for microarray analysis of defined tumor areas. *Biotechniques* **32:** (4) 744.

Davidsson P, Westman-Brinkmalm A, Nilsson CL, Lindbjer M, Paulson L, Andreasen N, Sjogren M, Blennow K. 2002. Univ Gothenburg, Sahlgrens Univ Hosp, Dept Clin Neurosci, SE-43180 Molndal, Sweden. Proteome analysis of cerebrospinal fluid in Alzheimer patients. *Neuroreport* **13:** (5) 611.

Dean PM, Zanders ED. 2002. De Novo Pharmaceut Ltd, St Andrews Hse, 59 St Andrews St, Cambridge CB2 3DD, England. The use of chemical design tools to transform proteomics data into drug candidates. *Biotechniques* (Suppl) 28.

Delpuech O, Trabut JB, Carnot F, Feuillard J, Brechot C, Kremsdorf D*. 2002. *Fac Med Necker Enfants Malad, INSERM U370, 156 rue Vaugirard, FR-75730 Paris 15, France. Identification, using cDNA macroarray analysis, of distinct gene expression profiles associated with pathological and virological features of hepatocellular carcinoma. *Oncogene* **21:** (18) 2926.

Gaiser T, Thorns C, Merz H, Noack F, Feller AC, Lange K*. 2002. *Med Univ, Dept Pathol, Ratzeburger Allee 160, DE-23538 Lubeck, Germany. Gene profiling in anaplastic large-cell lymphoma-derived cell lines with cDNA expression arrays. *J Hematother* **11:** (2) 423.

Golemis EA, Tew KD, Dadke D. 2002. Fox Chase Canc Ctr, 7701 Burholme Ave, Philadelphia, Pa 19111, USA. Protein interaction targeted drug discovery: Evaluating critical issues. *Biotechniques* **32:** (3) 636.

Gutmann DH, Hedrick NM, Li J, Nagarajan R, Perry A, Watson MA. 2002. Washington Univ, Dept Neurol, 660 Sth Euclid St, St Louis, Mo 63110, USA. Comparative gene expression profile analysis of neurofibromatosis 1-associated and sporadic pilocytic astrocytomas. *Cancer Res* **62:** (7) 2085.

Gutmann DH, Huang ZY, Hedrick NM, Ding H, Guha A, Watson MA. 2002. Address as above. Mouse glioma gene expression profiling identifies novel human glioma-associated genes. *Ann Neurol* **51:** (3) 393.

Haas G, Karaali G, Ebermayer K, Metzger WG, Lamer S, Zimny-Arndt U, Diescher S, Goebel UB, Vogt K, Roznowski AB *et al*. 2002. c/o Meyer TF, St Louis Univ, Div Endocrinol, 1402 Sth Grand Blvd, St Louis, Mo 63104, USA. Immunoproteomics of *Helicobacter pylori* infection and relation to gastric disease. *Proteomics* **2:** (3) 313.

Helfrich JP. 2002. Nugenesis Technol, Drug Discovery & Dev Grp, 1900 West Park Dr, Westborough, Ma 01581, USA. Raw data to knowledge warehouse in proteomic-based drug discovery: A scientific data management issue. *Biotechniques* (Suppl) 48.

Hernandez MR, Agapova OA, Yang P, Salvador-Silva M, Ricard CS, Aoi S. 2002. Washington Univ, Dept Ophthalmol & Visual Sci, 660 Sth Euclid Ave, St Louis, Mo 63110, USA. Differential gene expression in astrocytes from human normal and glaucomatous optic nerve head analyzed by cDNA microarray. *Glia* **38:** (1) 45.

Huber A, Yee C, Darling TN, Yancey KB*. 2002. *Med Coll Wisconsin, Dept Dermatol, 8701 Watertown Plank Rd, Milwaukee, Wi 53226, USA. Comprehensive analysis of gene expression profiles in keratinocytes from patients with generalized atrophic benign epidermolysis bullosa. *Exp Dermatol* **11:** (1) 75.

Jeoung D, Oh SJ, Lee SG, Baek MG, Lee YH, Baek NI, Kim HY*. 2002. *Kyung Hee Univ, Grad Sch Biotechnol, Suwon 449701, South Korea. Microarray analysis of piceatannol-induced changes in gene expression in human gastric cancer cells. *Biotechnol Lett* **24:** (6) 463.

Jiang YQ, Harlocker SL, Molesh DA, Dillon DC, Stolk JA, Houghton RL, Repasky EA, Badaro R, Reed SG, Xu JC. 2002. Corixa Corp, 1124 Columbia St, Seattle, Wa 98104, USA. Discovery of differentially expressed genes in human breast cancer using subtracted cDNA libraries and cDNA microarrays. *Oncogene* **21:** (14) 2270.

Kraggerud SM, Skotheim RI, Szymanska J, Eknaes M, Fossa SD, Stenwig AE, Peltomaki P, Lothe RA*. 2002. *Norwegian Radium Hosp, Inst Canc Res, Dept Genet, NO-0310 Oslo, Norway. Genome profiles of familial/bilateral and sporadic testicular germ cell tumors. *Genes Chromosomes Cancer* **34:** (2) 168.

Kroll T, Odyvanova L, Clement JH, Platzer C, Naumann A, Marr N, Hoffken K, Wolfl S*. 2002. *Univ Jena, Clin Internal Med Hematol Oncol Metab Dis & Diabet, Erlanger Allee 101, DE-07740 Jena, Germany. Molecular characterization of breast cancer cell lines by expression profiling. *J Cancer Res Clin Oncol* **128:** (3) 125.

Kumar Y, Uppuluri NRV, Babu K, Phadke K, Kumar P, Ballal S, Tatu U*. 2002. *Indian Inst Sci, Dept Biochem, IN-560012 Bangalore, India. Proteomics of renal disorders: Urinary proteome analysis by two-dimensional gel electrophoresis and MALDI-TOF mass spectrometry. *Curr Sci* **82:** (6) 655.

Li BR, Perabekam S, Liu G, Yin M, Song SW, Larson A. 2002. Case Western Reserve Univ, Dept Biochem, Wood Basic Sci Bldg, Cleveland, Oh 44106, USA. Experimental and bioinformatics comparison of gene expression between T-cells from TIL of liver cancer and T-cells from UniGene. *J Gastroenterol* **37:** (4) 275.

Li S, Xia X, Zhang X, Suen J. 2002. Henry Ford Hlth Syst, Dept Otolaryngol Head & Neck Surg, 2799 West Grand Blvd, Detroit, Mi 48202, USA. Regression of tumors by IFN-$\alpha$ electroporation gene therapy and analysis of the responsible genes by cDNA array. *Gene Ther* **9:** (6) 390.

Lim SO, Park SJ, Kim W, Park SG, Kim HJ, Kim YI, Sohn TS, Noh JH, Jung G*. 2002. *Seoul Natl Univ, Sch Biol Sci, Seoul 151747, South Korea. Proteome analysis of hepatocellular carcinoma. *Biochem Biophys Res Commun* **291:** (4) 1031.

Mousses S, Bubendorf L, Wagner U, Hostetter G, Kononen J, Cornelison R, Goldberger N, Elkahloun AG, Willi N, Koivisto P *et al*. 2002. c/o Kallioniemi OP, NIH/NHGRI, Canc Genet Branch, 50 Sth Dr, Bethesda, Md 20892, USA. Clinical validation of candidate genes associated with prostate cancer progression in the CWR22 model system using tissue microarrays. *Cancer Res* **62:** (5) 1256.

Naiki T, Nagaki M*, Shidoji Y, Kojima H, Imose M, Kato T, Ohishi N, Yagi K, Moriwaki H. 2002. *Gifu Univ, Dept Internal Med 1, 40 Tsukasa machi, Gifu 500 8705, Japan. Analysis of gene expression profile induced by hepatocyte nuclear factor 4$\alpha$ in hepatoma cells using an oligonucleotide microarray. *J Biol Chem* **277:** (16) 14011.

Nakajima H, Takenaka M*, Kaimori JY, Nagasawa Y, Kosugi A, Kawamoto S, Imai E, Hori M, Okubo K. 2002. *Osaka Univ, Dept Internal Med & Therapeut, 2-2 Yamadaoka, Suita, Osaka 565 0871, Japan. Gene-expression profile of renal proximal tubules regulated by proteinuria. *Kidney Int* **61:** (5) 1577.

Napoli C, De Nigris F, Welch JS, Calara FB, Stuart RO, Glass CK, Palinski W*. 2002. *Univ Calif San Diego, Dept Med, 9500 Gilman Dr, La Jolla, Ca 92093, USA. Maternal hypercholesterolemia during pregnancy promotes early atherogenesis in LDL receptor deficient mice and alters gene expression determined by microarray. *Circulation* **105:** (11) 1360.

Ning XH, Chen SH, Xu CS, Li LH, Yao LY, Qian K, Krueger JJ, Hyyti OM, Portman MA. 2002. Univ Washington, Div Cardiol, 1959 NE Pacific St, Seattle, Wa 98195, USA. Hypothermic protection of the ischemic heart *via* alterations in apoptotic pathways as assessed by gene array analysis. *J Appl Physiol* **92:** (5) 2200.

Qi ZY, Li Y, Ying K, Wu CQ, Tang R, Zhou ZX, Chen ZP, Hui GZ, Xie Y. 2002. Suzhou Univ, Affiliated Hosp 1, Dept Neurosurg, CN-215006 Suzhou, Peoples Rep China. Isolation of novel differentially expressed genes related to human glioma using cDNA microarray and characterizations of two novel full-length genes. *J Neurooncol* **56:** (3) 197.

Qin LF, Lee TKW, Ng IOL*. 2002. *Univ Hong Kong, Queen Mary Hosp, Dept Pathol, 102 Pokfulam Rd, Hong Kong, Peoples Rep China. Gene expression profiling by cDNA array in human hepatoma cell line in response to cisplatin treatment. *Life Sci* **70:** (14) 1677.

Copyright © 2002 John Wiley & Sons, Ltd. 

Rupp H, Maisch B. 2002. Univ Marburg, Mol Kardiol Lab, Karl Frisch Str 1, DE-35033 Marburg, Germany. Functional genomics of the pressure loaded cardiomyocyte: Is heart failure a target for etomoxir? (German, English Abstract). *Herz* **27:** (2) 166.

Sardi I, Tintori V, Marchi C, Veltroni M, Lippi A, Tucci F, Tamburini A, Bernini G, Faulkner L. 2002. Univ Florence, Osped Pediat A Meyer, Unita Oncoematol, IT-50132 Florence, Italy. Molecular profiling of high-risk neuroblastoma by cDNA array. *Int J Mol Med* **9:** (5) 541.

Scanlan MJ, Gordon CM, Williamson B, Lee SY, Chen YT, Stockert E, Jungbluth A, Ritter G, Jager D, Jager E, Knuth A, Old LJ. 2002. Ludwig Inst Canc Res, Mem Sloan Kettering Canc Ctr, 1275 York Ave, New York, NY 10021, USA. Identification of cancer/testis genes by database mining and mRNA expression analysis. *Int J Cancer* **98:** (4) 485.

Tan C, Li J, Wang JR, Xiang Q, Zhang XM, Dong L, Shen SR, Liang SP, Li GY*. 2002. *Cent Sth Univ, Canc Res Inst, 88 Xiangya Rd, CN-410078 Changsha, Hunan, Peoples Rep China. Proteomic analysis of differential protein expression in human nasopharyngeal carcinoma cells induced by NAG7 transfection. *Proteomics* **2:** (3) 306.

Tan S, Seow TK, Liang RCMY, Koh S, Lee CPC, Chung MCM, Hooi SC*. 2002. *Natl Univ Singapore, Dept Physiol, 2 Med Dr, SG-117597 Singapore, Rep Singapore. Proteome analysis of butyrate-treated human colon cancer cells (HT-29). *Int J Cancer* **98:** (4) 523.

Wahde M, Klus GT, Bittner ML, Chen YD, Szallasi Z. 2002. Chalmers Univ Technol, Div Mechatronics, SE-41296 Gothenburg, Sweden. Asessing the significance of consistently mis-regulated genes in cancer associated gene expression matrices. *Bioinformatics* **18:** (3) 389.

Wellmann A, Wollscheid V, Lu H, Ma ZL, Albers P, Schutze K, Rohde V, Behrens P, Dreschers S, Ko Y, Wernert N. 2002. Univ Bonn, Inst Pathol, Sigmund Freud Str 25, DE-53127 Bonn, Germany. Analysis of microdissected prostate tissue with ProteinChip® arrays: A way to new insights into carcinogenesis and to diagnostic tools. *Int J Mol Med* **9:** (4) 341.

Zhao JM, Roth J, Bode-Lesniewska B, Pfaltz M, Heitz PU, Komminoth P. 2002. Univ Zurich, Dept Pathol, Schmelzbergstr 12, CH-8091 Zurich, Switzerland. Combined comparative genomic hybridization and genomic microarray for detection of gene amplifications in pulmonary artery intimal sarcomas and adrenocortical tumors. *Gene Chromosomes Cancer* **34:** (1) 48.

### 8 EST, cDNA and other clone resources

Buraczynska M, Mears AJ, Zareparsi S, Farjo R, Filippova E, Yuan YK, MacNee SP, Hughes B, Swaroop A*. 2002. *Univ Michigan, Dept Opthalmol & Visual Sci, 1000 Wall St, Ann Arbor, Mi 48105, USA. Gene expression profile of native human retinal pigment epithelium. *Invest Ophthalmol Vis Sci* **43:** (3) 603.

Channeliere S, Riviere S, Scalliet G, Szecsi J, Jullien F, Dolle C, Vergne P, Dumas C, Bendahmane M, Hugueney P, Cock JM*. 2002. *ENSL, UMR 5667 CNRS-INRA, 46 allee d'Italie, FR-69364 Lyon 07, France. Analysis of gene expression in rose petals using expressed sequence tags. *FEBS Lett* **515:** (1-3) 35.

Fernandes J, Brendel V, Gai XW, Lal S, Chandler VL, Elumalai P, Galbraith DW, Pierson EA, Walbot V*. 2002. *Stanford Univ, Dept Biol Sci, Stanford, Ca 94305, USA. Comparison of RNA expression profiles based on maize expressed sequence tag frequency analysis and micro-array hybridization. *Plant Physiol* **128:** (3) 896.

Kantety RV, La Rota M, Matthews DE, Sorrells ME*. 2002. *Cornell Univ, Dept Plant Breeding, 252 Emerson Hall, Ithaca, NY 14853, USA. Data mining for simple sequence repeats in expressed sequence tags from barley, maize, rice, sorghum and wheat. *Plant Mol Biol* **48:** (5) 501.

Lee S, Clark T, Chen JJ, Zhou GL, Scott LR, Rowley JD, Wang SM*. 2002. *Univ Chicago, Dept Med, 5841 Sth Maryland Ave, Chicago, Il 60637, USA. Correct identification of genes from serial analysis of gene expression tag sequences. *Genomics* **79:** (4) 598.

Shoemaker R, Keim P, Vodkin L, Retzel E, Clifton SW, Waterston R, Smoller D, Coryell V, Khanna A, Erpelding J *et al.* 2002. Iowa State Univ, USDA/ARS, Corn Insect & Crop Genet Res Unit, Ames, Ia 50011, USA. A compilation of soybean ESTs: Generation and analysis. *Genome* **45:** (2) 329.

Smith EJ, Shi L, Smith G. 2002. Virginia Polytech Inst & State Univ, Dept Anim & Poultry Sci, Blacksburg, Va 24061, USA. Expressed sequence tags for the chicken genome from a normalized 10-day-old white leghorn whole-embryo cDNA library. III. DNA sequence analysis of genetic variation in commercial chicken populations. *Genome* **45:** (2) 261.

Whitefield CW, Band MR, Bonaldo MF, Kumar CG, Liu L, Pardinas JR, Robertson HM, Soares MB, Robinson GE*. 2002. *Univ Illinois, Dept Entomol, Urbana, Il 61801, USA. Annotated expressed sequence tags and cDNA microarrays for studies of brain and behavior in the honey bee. *Genome Res* **12:** (4) 555.

### 9 Functional genomics

Consoli L, Lefevre A, Zivy M, De Vienne D, Damerval C*. 2002. *INRA/INA-PG/UPS, Stn Genet Vegetale, FR-91190 Gif-sur-Yvette, France. QTL analysis of proteome and transcriptome variations for dissecting the genetic architecture of complex traits of maize. *Plant Mol Biol* **48:** (5) 575.

Endo M, Matsubara H, Kokubun T, Masuko H, Takahata Y, Tsuchiya T, Fukuda H, Demura T, Watanabe M*. 2002. *Iwate Univ, Lab Plant Breeding, 3-18-8 Ueda, Morioka, Iwate 020 8550, Japan. The advantages of cDNA microarray as an effective tool for identification of reproductive organ-specific genes in a model legume, *Lotus japonicus*. *FEBS Lett* **514:** (2-3) 229.

Forsyth RA, Haselbeck RJ, Ohlsen KL, Yamamoto RT, Xu H, Trawick JD, Wall D, Wang L, Brown-Driver V, Froelich JM *et al.* 2002. c/o Foulkes JG, Elitra Pharmaceut, San Diego, Ca 92121, USA. A genome-wide strategy for the identification of essential genes in *Staphylococcus aureus*. *Mol Microbiol* **43:** (6) 1387.

Huang W, Sher YP, Peck K, Fung YCB*. 2002. *Univ Calif San Diego, Dept Bioengn, La Jolla, Ca 92093, USA. Matching gene activity with physiological functions. *Proc Natl Acad Sci U S A* **99:** (5) 2603.

Jun JH, Kim CS, Cho DS, Kwak JM, Ha CM, Park YS, Cho BH, Patton DA, Nam HG*. 2002. *Univ Sci & Technol, Div Mol Life Sci, Pohang 790784, Kyungbuk, South Korea. Random antisense cDNA mutagenesis as an efficient functional genomic approach in higher plants. *Planta* **214:** (5) 668.

Liang P, Labedan B, Riley M. 2002. Roswell Pk Canc Inst, Dept Canc Genet, Elm & Carlton St, Buffalo, NY 14263, USA. Physiological genomics of *Escherichia coli* protein families. *Physiol Genomics* **9:** (1) 15.

Lukacsovich T, Yamamoto D. 2001. Waseda Univ, Sch Human Sci, 2-579-15 Mikajima, Tokorozawa, Saitama 359 1192, Japan. Trap a gene and find out its function: Toward functional genomics in *Drosophila*. *J Neurogenet* **15:** (3-4) 147.

Nagel AC, Maier D, Preiss A*. 2002. *Univ Hohenheim, Inst Genet, Garbenstr 30, DE-70599 Stuttgart, Germany. Green fluorescent protein as a convenient and versatile marker for studies on functional genomics in *Drosophila*. *Dev Genes Evol* **212:** (2) 93.

Ramet M, Manfruelli P, Pearson A, Mathey-Prevot B, Ezekowitz RAB. 2002. MGH Children, Lab Dev Immunol, 55 Fruit St, Boston, Ma 02114, USA. Functional genomic analysis of phagocytosis and identification of a *Drosophila* receptor for *E. coli*. *Nature* **416:** (6881) 644.

Reddy VS, Ali GS, Reddy ASN*. 2002. *Colorado State Univ, Dept Biol, Fort Collins, Co 80523, USA. Genes encoding calmodulin-binding proteins in the *Arabidopsis* genome. *J Biol Chem* **277:** (12) 9840.

Seki M, Narusaka M, Kamiya A, Ishida J, Satou M, Sakurai T, Nakajima M, Enju A, Akiyama K, Oono Y *et al.* 2002. c/o Shinozaki K, RIKEN, Tsukuba Inst, Plant Mol Biol Lab, 3-1-1 Koyadai, Tsukuba, Ibaraki 305 0074, Japan. Functional annotation of a full-length *Arabidopsis* cDNA collection. *Science* **296:** (5565) 141.

Shusta EV, Boado RJ, Mathern GW, Pardridge WM*. 2002. *UCLA, Dept Med, 900 Veterans Avenue, Los Angeles, Ca 90024, USA. Vascular genomics of the human brain. *J Cereb Blood Flow Metab* **22:** (3) 245.

Wen ZT, Burne RA*. 2002. *Univ Florida, Dept Oral Biol, Box 100424, Gainesville, Fl 32610, USA. Functional genomics approach to identifying genes required for biofilm development by *Streptococcus mutans*. *Appl Environ Microbiol* **68:** (3) 1196.

Williams RM. 2002. Diversa, 4995 Directors Pl, San Diego, Ca 92121, USA. The yeast lifecycle and DNA array technology. *J Ind Microbiol Biotechnol* **28:** (3) 186.

Copyright © 2002 John Wiley & Sons, Ltd. 

### 10 Transcriptomics

Akhtar RA, Reddy AB, Maywood ES, Clayton JD, King VM, Smith AG, Gant TW, Hastings MH, Kyriacou CP*. 2002. *Univ Leicester, Dept Genet, Univ Rd, Leicester LE1 7RH, England. Circadian cycling of the mouse liver transcriptome, as revealed by cDNA microarray, is driven by the suprachiasmatic nucleus. *Curr Biol* **12:** (7) 540.

Chan JYH, Chen LK, Chang JF, Ting HM, Goy C, Chen JL, Hwang JJ, Chen FD, Chen DJ, Ngo FQH*. 2001. *Natl Yang Ming Univ, Inst Radiol Sci, Taipei 112, Taiwan. Differential gene expression in a DNA double-strand-break repair mutant XRS-5 defective in ku80: Analysis by cDNA microarray. *J Radiat Res* **42:** (4) 371.

Chen WQ, Provart NJ, Glazebrook J, Katagiri F, Chang HS, Eulgem T, Mauch F, Luan S, Zou GZ, Whitham SA *et al*. 2002. c/o Zhu T, Syngenta Res & Technol, 3115 Merryfield Row, San Diego, Ca 92121, USA. Expression profile matrix of *Arabidopsis* transcription factor genes suggests their putative functions in response to environmental stresses. *Plant Cell* **14:** (3) 559.

Clarkson MR, Murphy M, Gupta S, Lambe T, Mackenzie HS, Godson C, Martin F, Brady HR*. 2002. *Univ Coll Dublin, Mater Misericordiae Hosp, Dept Med & Therapeut, 41 Eccles St, Dublin 7, Rep Ireland. High glucose-altered gene expression in mesangial cells: Actin-regulatory protein gene expression is triggered by oxidative stress and cytoskeletal disassembly. *J Biol Chem* **277:** (12) 9707.

Dougherty JD, Geschwind DH*. 2002. *UCLA, Reed Neurol Res Ctr, 710 Westwood Plaza, Los Angeles, Ca 90095, USA. Subtraction-coupled custom microarray analysis for gene discovery and gene expression studies in the CNS. *Chem Senses* **27:** (3) 293.

Dozmorov I, Galecki A, Chang YY, Krzesicki R, Vergara M, Miller RA*. 2002. *Univ Michigan, Dept Pathol, Box 0940, Ann Arbor, Mi 48109, USA. Gene expression profile of long-lived Snell dwarf mice. *J Gerontol A-Biol Sci Med* **57:** (3) B99.

Duffield GE, Best JD, Meurers BH, Bittner A, Loros JJ, Dunlap JC. 2002. Dartmouth Coll Sch Med, Dept Genet, Hanover, NH 03755, USA. Circadian programs of transcriptional activation, signaling, and protein turnover revealed by microarray analysis of mammalian cells. *Curr Biol* **12:** (7) 551.

Eymann C, Homuth G, Scharf C, Hecker M*. 2002. *Univ Greifswald, Inst Mikrobiol, FL Jahn Str 5, DE-17487 Greifswald, Germany. *Bacillus subtilis* functional genomics: Global characterization of the stringent response by proteome and transcriptome analysis. *J Bacteriol* **184:** (9) 2500.

Guckenberger M, Kurz S, Aepinus C, Theiss S, Haller S, Leimbach T, Panzner U, Weber J, Paul H, Unkmeir A *et al*. 2002. c/o Frosch M, Univ Wurzburg, Inst Hyg & Microbiol, Josef Schneider Str 2, DE-97080 Wurzburg, Germany. Analysis of the heat shock response of *Neisseria meningitidis* with cDNA- and oligonucleotide-based DNA microarrays. *J Bacteriol* **184:** (9) 2546.

Hu L, Cogdell DE, Jia YJ, Hamilton SR, Zhang W*. 2002. *Univ Texas, Canc Genomics Core Lab, MD Anderson Canc Ctr, Houston, Tx 77030, USA. Monitoring of cDNA microarray with common primer target and hybridization specificity with selected targets. *Biotechniques* **32:** (3) 528.

Karsi A, Cao D, Li P, Patterson A, Kocabas A, Feng J, Ju Z, Mickett KD, Liu Z*. 2002. *Auburn Univ, Dept Fisheries & Allied Aquacultures, Auburn, Al 36849, USA. Transcriptome analysis of channel catfish (*Ictalurus punctatus*): Initial analysis of gene expression and micro satellite-containing cDNAs in the skin. *Gene* **285:** (1-2) 157.

Klein CA, Seidl S, Petat-Dutter K, Offner S, Geigl JB, Schmidt-Kittler O, Wendler N, Passlick B, Huber RM, Schlimok G *et al*. 2002. Univ Munich, Klinikum Innenstadt, Inst Immunol, DE-80336 Munich, Germany. Combined transcriptome and genome analysis of single micrometastatic cells. *Nat Biotechnol* **20:** (4) 387.

Lorenz WW, Dean JFD*. 2002. *Univ Georgia, DB Warnell Sch Forest Resources, Athens, Ga 30602, USA. SAGE profiling and demonstration of differential gene expression along the axial developmental gradient of lignifying xylem in loblolly pine (*Pinus taeda*). *Tree Physiol* **22:** (5) 301.

Marvin KW, Keelan JA, Eykholt RL, Sato TA, Mitchell MD. 2002. Univ Auckland, Fac Med & Hlth Sci, 85 Pk Rd, Auckland 1, New Zealand. Use of cDNA arrays to generate differential expression profiles for inflammatory genes in human gestational membranes delivered at term and preterm. *Mol Hum Reprod* **8:** (4) 399.

Ohki R, Yamamoto K*, Mano H, Lee RT, Ikeda U, Shimada K. 2002. *Jichi Med Sch, Dept Cardiol, Minami Kawachi, Tochigi 329 0498, Japan. Identification of mechanically induced genes in human monocytic cells by DNA microarrays. *J Hypertens* **20:** (4) 685.

Ozturk ZN, Talame V, Deyholos M, Michalowski CB, Galbraith DW, Gozukirmizi N, Tuberosa R, Bohnert HJ*. 2002. *Univ Arizona, Dept Biochem & Mol Biophys, Tucson, Az 85721, USA. Monitoring large-scale changes in transcript abundance in drought- and salt-stressed barley. *Plant Mol Biol* **48:** (5) 551.

Raouf A, Seth A*. 2002. *Univ Toronto, Dept Lab Med & Pathobiol, 1 Taddle Creek Rd, Toronto, Ontario, Canada M5S 1A8. Discovery of osteoblast-associated genes using cDNA microarrays. *Bone* **30:** (3) 463.

Scheideler M, Schlaich NL*, Fellenberg K, Beissbarth T, Hauser NC, Vingron M, Slusarenko AJ, Hoheisel JD. 2002. *Rhein Westfal TH Aachen, Inst Pflanzenphysiol, Worringerweg 1, DE-52074 Aachen, Germany. Monitoring the switch from housekeeping to pathogen defense metabolism in *Arabidopsis thaliana* using cDNA arrays. *J Biol Chem* **277:** (12) 10555.

Shedden K, Cooper S*. 2002. *Univ Michigan, Dept Stat, Ann Arbor, Mi 48109, USA. Analysis of cell-cycle-specific gene expression in human cells as determined by microarrays and double-thymidine block synchronization. *Proc Natl Acad Sci U S A* **99:** (7) 4379.

Su AI, Cooke MP, Ching KA, Hakak Y, Walker JR, Wiltshire T, Orth AP, Vega RG, Sapinoso LM, Moqrich A *et al*. 2002. c/o Hogenesch JB, Novartis Res Fdn, Genomics Inst, 3115 Merryfield Row, San Diego, Ca 92121, USA. Large-scale analysis of the human and mouse transcriptomes. *Proc Natl Acad Sci U S A* **99:** (7) 4465.

Takeuchi H, Fujiyuki T, Shirai K, Matsuo Y, Kamikouchi A, Fujinawa Y, Kato A, Tsujimoto A, Kubo T*. 2002. *Univ Tokyo, Dept Sci Biol, Bunkyo ku, Tokyo 113 0033, Japan. Identification of genes expressed preferentially in the honeybee bodies by combination of differential display and cDNA microarray. *FEBS Lett* **513:** (2-3) 230.

Ueda HR, Matsumoto A, Kawamura M, Iino M, Tanimura T, Hashimoto S. 2002. Univ Tokyo, Dept Pharmacol, Bunkyo ku, Tokyo 1130033, Japan. Genome-wide transcriptional orchestration of circadian rhythms in *Drosophila*. *J Biol Chem* **277:** (16) 14048.

### 11 Proteomics

Braun P, Hu YH, Shen BH, Halleck A, Koundinya M, Harlow E, La Baer J*. 2002. *Harvard Univ, Inst Proteomics, 240 Longwood Ave, Boston, Ma 02115, USA. Proteome-scale purification of human proteins from bacteria. *Proc Natl Acad Sci U S A* **99:** (5) 2654.

Chakraborty A, Regnier FE*. 2002. *Purdue Univ, Dept Chem, West Lafayette, In 47907, USA. Global internal standard technology for comparative proteomics. *J Chromatogr A* **949:** (1-2) 173.

Cobon GS, Verrills N, Papakostopoulos P, Eastwood H, Linnane AW. 2002. Macquarie Univ, Australian Proteome Anal Facil, Sydney, NSW, Australia. The proteomics of ageing. *Biogerontology* **3:** (1-2) 133.

Eriksson J, Fenyo D*. 2002. *Proteometrics LLC, 7 West 36th St, New York, NY 10018, USA. A model of random mass-matching and its use for automated significance testing in mass spectrometric proteome analysis. *Proteomics* **2:** (3) 262.

Fratelli M, Demol H, Puype M, Casagrande S, Eberini I, Salmona M, Bonetto V, Mengozzi M, Duffieux F, Miclet E *et al*. 2002. c/o Ghezzi P, Mario Negri Inst Pharmacol Res, via Eritrea 62, IT-20157 Milan, Italy. Identification by redox proteomics of glutathionylated proteins in oxidatively stressed human T-lymphocytes. *Proc Natl Acad Sci U S A* **99:** (6) 3505.

Fung ET, Enderwick C. 2002. Ciphergen Biosyst, 6611 Dumbarton Circle, Fremont, Ca 94555, USA. ProteinChip® clinical proteomics: Computational challenges and solutions. *Biotechniques* (Suppl) 34.

Graslund S, Falk R, Brundell E, Hoog C, Stahl S*. 2002. *Royal Inst Technol, Dept Biotechnol, SE-10691 Stockholm, Sweden. A high-stringency proteomics concept aimed for generation of antibodies specific for cDNA-encoded proteins. *Biotechnol Appl Biochem* **35:** (2) 75.

Harrison P, Kumar A, Lan N, Echols N, Snyder M, Gerstein M*. 2002. *Yale Univ, Dept Mol Biophys & Biochem, 266 Whitney Ave, New Haven, Ct 06520, USA. A small reservoir of disabled ORFs in the yeast genome and its implications for the dynamics of proteome evolution. *J Mol Biol* **316:** (3) 409.

Hernandez-Macedo ML, Ferraz A, Rodriguez J, Ottoboni LMM, De

Copyright © 2002 John Wiley & Sons, Ltd.

Mello MP*. 2002. *UNICAMP, CBMEG, CP 6010, BR-13083-970 Campinas, SP, Brazil. Iron-regulated proteins in *Phanerochaete chrysosporium* and *Lentinula edodes*: Differential analysis by sodium dodecyl sulfate polyacrylamide gel electrophoresis and two-dimensional polyacrylamide gel electrophoresis profiles. *Electrophoresis* **23:** (4) 655.

Isfort RJ, Wang F, Greis KD, Sun YP, Keough TW, Bodine SC, Anderson NL. 2002. Procter & Gamble Pharmaceut, 8700 Mason Montgomery Rd, Mason, Oh 45040, USA. Proteomic analysis of rat soleus and tibialis anterior muscle following immobilization. *J Chromatogr B* **769:** (2) 323.

Kanamoto T, Hellman U, Heldin CH, Souchelnytskyi S*. 2002. *Ludwig Inst Canc Res, Box 595, SE-75124 Uppsala, Sweden. Functional proteomics of transforming growth factor-β1-stimulated Mv1Lu epithelial cells: Rad51 as a target of TGFβ1-dependent regulation of DNA repair. *EMBO J* **21:** (5) 1219.

Klose J, Nock C, Herrmann M, Stuhler K, Marcus K, Bluggel M, Krause E, Schalkwyk LC, Rastan S, Brown SDM *et al.* 2002. Inst Humangenet, Campus Virchow Klinikum, Augustenburger Pl 1, DE-13353 Berlin, Germany. Genetic analysis of the mouse brain proteome. *Nat Genet* **30:** (4) 385.

Lehr S, Kotzka J, Knebel B, Schiller M, Krone W, Muller-Wieland D. 2002. Univ Dusseldorf, Abt Klin Biochem & Pathobiochem, Hennekamp 65, DE-40225 Dusseldorf, Germany. Primary skin fibroblasts as human model system for proteome analysis. *Proteomics* **2:** (3) 280.

Liu HB, Lin DY, Yates JR*. 2002. *Scripps Clin & Res Inst, Dept Cell Biol, La Jolla, Ca 92037, USA. Multidimensional separations for protein/peptide analysis in the post-genomic era. *Biotechniques* **32:** (4) 898.

Lopez MF, Melov S. 2002. Proteome Syst, 14 Gill St, Woburn, Ma 01801, USA. Applied proteomics: Mitochondrial proteins and effect on function. *Circ Res* **90:** (4) 380.

Moura H, Visvesvara GS. 2001. CDCP, Atlanta Res & Educ Fdn, 4770 Buford Highway NE, Chamblee, Ga 30341, USA. A proteome approach to the host-parasite interaction of the microsporidian encephalitozoon intestinalis. *J Eukaryot Microbiol* (Suppl) S56.

O'Neill EE, Brock CJ, Von Kriegsheim AF, Pearce AC, Dwek RA, Watson SP, Hebestreit HF*. 2002. *Univ Oxford, Oxford Glyobiol Inst, Sth Parks Rd, Oxford OX1 3QU, England. Towards complete analysis of the platelet proteome. *Proteomics* **2:** (3) 288.

Ohi MD, Link AJ, Ren LP, Jennings JL, McDonald WH, Gould KL*. 2002. *Vanderbilt Univ, Dept Cell Biol, Nashville, Tn 37232, USA. Proteomics analysis reveals stable multiprotein complexes in both fission and budding yeasts containing Myb-related Cdc5p/Cef1p, novel pre-mRNA splicing factors, and snRNAs. *Mol Cell Biol* **22:** (7) 2011.

Rao JY, Seligson D, Hemstreet GP. 2002. UCLA, Dept Pathol & Lab Med, 10833 Le Conte Ave, Los Angeles, Ca 90095, USA. Protein expression analysis using quantitative fluorescence image analysis on tissue microarray slides. *Biotechniques* **32:** (4) 924.

Richards P, Lees J. 2002. MRC Toxicol Unit, Hodgkin Bldg, Lancaster Rd, Leicester LE1 9HN, England. Functional proteomics using microchannel plate detectors. *Proteomics* **2:** (3) 256.

Rubenwolf S, Niewohner J, Meyer E, Petit-Frere C, Rudert F, Hoffmann PR, Ilag LL. 2002. Xerion Pharmaceut AG, Fraunhoferstr 9, DE-82152 Martinsried, Germany. Functional proteomics using chromophore-assisted laser inactivation. *Proteomics* **2:** (3) 241.

Saalbach G, Erik P, Wienkoop S. 2002. Riso Natl Lab, Dept Plant Res, POB 49, DK-4000 Roskilde, Denmark. Characterisation by proteomics of peribacteroid space and peribacteroid membrane preparations from pea (*Pisum sativum*) symbiosomes. *Proteomics* **2:** (3) 325.

Schubert M, Petersson UA, Haas BJ, Funk C, Schroder WP*, Kieselbach T. 2002. *Karolinska Inst, Dept Biosci, Novum, SE-14186 Huddinge, Sweden. Proteome map of the chloroplast lumen of *Arabidopsis thaliana*. *J Biol Chem* **277:** (10) 8354.

Schweitzer B, Roberts S, Grimwade B, Shao W, Wang M, Fu Q, Shu Q, Laroche I, Zhou Z, Tchernev VT *et al.* 2002. c/o Kingsmore SF, Protometrix Inc, 66 High St, Guilford, Ct 06437, USA. Multiplexed protein profiling on microarrays by rolling-circle amplification. *Nat Biotechnol* **20:** (4) 359.

Wang SH, Zhang X, Regnier FE*. 2002. *Purdue Univ, Dept Chem, West Lafayette, In 47907, USA. Quantitative proteomics strategy involving the selection of peptides containing both cysteine and histidine from tryptic digests of cell lysates. *J Chromatogr A* **949:** (1-2) 153.

### 12 Protein structural genomics

Aloy P, Oliva B, Querol E, Aviles FX, Russell RB*. 2002. *EMBL Meyerhofstr 1, DE-69117 Heidelberg, Germany. Structural similarity to link sequence space: New potential superfamilies and implications for structural genomics. *Protein Sci* **11:** (5) 1101.

Douguet D, Bolla JM, Munier-Lehmann H, Labesse G*. 2002. *Univ Montpellier I, Ctr Biochim Struct, UMR 5048 CNRS, U554 INSERM, 15 Ave Flahault, FR-34060 Montpellier 2, France. From sequence to structure to function: A case study. *Enzyme Microb Technol* **30:** (3) 289.

Frishman D. 2002. GSF, Natl Res Ctr Environm & Hlth, Inst Bioinformatics, Ingolstadter Landstr 1, DE-85764 Neuherberg, Germany. Knowledge-based selection of targets for structural genomics. *Protein Eng* **15:** (3) 169.

Mylvaganam SE, Prabhakaran M, Tudor SS, Moezzi S, Ramnarayan K*. 2002. *Struct Bioinformat, 10929 Technol Pl, San Diego, Ca 92127, USA. Structural proteomics: Methods in deriving protein structural information and issues in data management. *Biotechniques* (Suppl) 42.

Terashima H, Fukuchi S, Nakai K, Arisawa M, Hamada K, Yabuki N, Kitada K*. 2002. *Nippon Roche Res Ctr, Dept Mycol, 200 Kajiwara, Kamakura, Kanagawa 247 853, Japan. Sequence-based approach for identification of cell wall proteins in *Saccharomyces cerevisiae*. *Curr Genet* **40:** (5) 311.

Xu Y, Xu D, Olman V. 2002. Oak Ridge Natl Lab, Protein Informat Grp, 1060 Commerce Pk Dr, Oak Ridge, Tn 37830, USA. A practical method for interpretation of threading scores: An application of neural network. *Stat Sinica* **12:** (1) 159.

### 13 Metabolomics

Chambergo FS, Bonaccorsi ED, Ferreira AJS, Ramos ASP, Junior JRF, Abrahao-Neto J, Farah JPS, El Dorry H*. 2002. *Univ Sao Paulo, Dept Biochem, Avda Prof Lineu Prestes 748, BR-05508-900 Sao Paulo, Brazil. Elucidation of the metabolic fate of glucose in the filamentous fungus *Trichoderma reesei* using expressed sequence tag (EST) analysis and cDNA microarrays. *J Biol Chem* **277:** (16) 13983.

Kierzek AM. 2002. Polish Acad Sci, Inst Biochem & Biophys, Pawinskiego 5A, PL-02106 Warsaw, Poland. STOCKS: STOChastic Kinetic Simulations of biochemical systems with Gillespie algorithm. *Bioinformatics* **18:** (3) 470.

Middleton FA, Mirnics K, Pierri JN, Lewis DA, Levitt P*. 2002. *Univ Pittsburgh, Dept Neurobiol, 3500 Terrace St, Pittsburgh, Pa 15261, USA. Gene expression profiling reveals alterations of specific metabolic pathways in schizophrenia. *J Neurosci* **22:** (7) 2718.

Oh MK, Rohlin L, Kao KC, Liao JC*. 2002. *UCLA, Dept Chem Engn, 5531 Boelter Hall, Los Angeles, Ca 90095, USA. Global expression profiling of acetate-grown *Escherichia coli*. *J Biol Chem* **277:** (15) 13175.

### 14 Genomic approaches to development

Brody T, Stivers C, Nagle J, Odenwald WF*. 2002. *NIH/NINCDS, Neurogenet Unit, Rockville Pike, Bethesda, Md 20892, USA. Identification of novel *Drosophila* neural precursor genes using a differential embryonic head cDNA screen. *Mech Dev* **113:** (1) 41.

Chauhan BK, Reed NA, Zhang WJ, Duncan MK, Kilimann MW, Cvekl A*. 2002. *Albert Einstein Coll Med, Dept Ophthalmol & Visual Sci, 1300 Morris Pk Ave, Bronx, NY 10461, USA. Identification of genes downstream of Pax6 in the mouse lens using cDNA microarrays. *J Biol Chem* **277:** (13) 11539.

Cui LW, Rzomp KA, Fan Q, Martin SK, Williams J. 2001. Penn State Univ, Dept Entomol, 501 ASI Bldg, University Park, Pa 16802, USA. *Plasmodium falciparum*: Differential display analysis of gene expression during gametocytogenesis. *Exp Parasitol* **99:** (4) 244.

Farjo R, Yu JD, Othman MI, Yoshida S, Sheth S, Glaser T, Baehr W, Swaroop A*. 2002. *Univ Michigan, Dept Ophthalmol, 1000 Wall St, Ann Arbor, Mi 48105, USA. Mouse eye gene microarrays for investigating ocular development and disease. *Vision Res* **42:** (4) 463.

Ohta S, Fuse H, Tabuchi Y. 2002. Med & Pharmaceut Univ, Dept Urol, 2630 Sugitani, Toyama 930 0194, Japan. DNA microarray analysis of genes involved in the process of differentiation in mouse Leydig cell

Copyright © 2002 John Wiley & Sons, Ltd. 

line TTE1. *Arch Androl* **48:** (3) 203.

Prolla TA. 2002. Univ Wisconsin, Dept Genet, 445 Henry Mall, Madison, Wi 53706, USA. DNA microarray analysis of the aging brain. *Chem Senses* **27:** (3) 299.

Rajkovic A, Matzuk MM*. 2002. *Baylor Coll Med, Dept Pathol, 1 Baylor Plaza, Houston, Tx 77030, USA. Functional analysis of oocyte-expressed genes using transgenic models. *Mol Cell Endocrinol* **187:** (1-2) 5.

Van Driessche N, Shaw C, Katoh M, Morio T, Sucgang R, Ibarra M, Kuwayama H, Saito T, Urushihara H, Maeda M *et al*. 2002. c/o Shaulsky G, Baylor Coll Med, Dept Mol & Human Genet, Houston, Tx 77030, USA. A transcriptional profile of multicellular development in *Dictyostelium discoideum*. *Development* **129:** (7) 1543.

Zhao Q, Kho A, Kenney AM, Yuk DI, Kohane I, Rowitch DH*. 2002. *Dana Farber Canc Inst, Dept Pediat Oncol, 44 Binney St, Boston, Ma 02115, USA. Identification of genes expressed with temporal-spatial restriction to developing cerebellar neuron precursors by a functional genomic approach. *Proc Natl Acad Sci U S A* **99:** (8) 5704.

### 15 Technological advances

Bao P, Frutos AG, Greef C, Lahiri J*, Muller U, Peterson TC, Warden L, Xie XY. 2002. *Corning Inc, Div Sci & Technol, Corning, NY 14831, USA. High-sensitivity detection of DNA hybridization on microarrays using resonance light scattering. *Anal Chem* **74:** (8) 1792.

Beck S, Sakurai T, Eustace BK, Beste G, Schier R, Rudert F, Jay DG*. 2002. *Tufts Univ Med, Dept Physiol, 136 Harrison Ave, Boston, Ma 02115, USA. Fluorophore-assisted light inactivation: A high-throughput tool for direct target validation of proteins. *Proteomics* **2:** (3) 247.

Berggren WT, Takova T, Olson MC, Eis PS, Kwiatkowski RW, Smith LM. 2002. Univ Wisconsin, Dept Chem, 1101 Univ Ave, Madison, Wi 53706, USA. Multiplexed gene expression analysis using the invader RNA assay with MALDI-TOF mass spectrometry detection. *Anal Chem* **74:** (8) 1745.

Bossio RE, Marshall AG*. 2002. *Florida State Univ, Natl High Magnet Field Lab, Tallahassee, Fl 32310, USA. Baseline resolution of isobaric phosphorylated and sulfated peptides and nucleotides by electrospray ionization FTICR MS: Another step toward mass spectrometry-based proteomics. *Anal Chem* **74:** (7) 1674.

Cho JC, Tiedje JM*. 2002. *Michigan State Univ, Ctr Microbial Ecol, East Lansing, Mi 48824, USA. Quantitative detection of microbial genes by using DNA microarrays. *Appl Environ Microbiol* **68:** (3) 1425.

Dederich DA, Okwuonu G, Garner T, Denn A, Sutton A, Escotto M, Martindale A, Delgado O, Muzny DM, Gibbs RA, Metzker ML*. 2002. *Baylor Coll Med, Human Genome Sequencing Ctr, 1 Baylor Plaza, Houston, Tx 77030, USA. Glass bead purification of plasmid template DNA for high throughput sequencing of mammalian genomes. (Online art. no. E32). *Nucleic Acids Res* **30:** (7) E32.

Egelhofer V, Gobom J, Seitz H, Giavalisco P, Lehrach H, Nordhoff E. 2002. Scienion AG, Volmerstr 7B, DE-12489 Berlin, Germany. Protein identification by MALDI-TOF-MS peptide mapping: A new strategy. *Anal Chem* **74:** (8) 1760.

Ettinger A, Ostroff R, Brien J, Polisky B. 2002. BioStar Inc, 6655 Lookout Rd, Boulder, Co 80301, USA. In-process monitoring of protein purification with thin film silicon sensor technology. *Biotechniques* **32:** (4) 934.

Hancock WS, Choudhary G, Wu SL, Shieh P. 2002. Thermo Finnigan, 355 River Oaks Rd, San Jose, Ca 95134, USA. A high-throughput solution for proteomics. *Am Lab* **34:** (5) 13.

Hu YX, Wang ZK, Wang YH, Bao F, Li N, Peng ZH, Li JY*. 2001. *Chinese Acad Sci, Inst Genet, CN-100101 Beijing, Peoples Rep China. Identification of brassinosteroid responsive genes in *Arabidopsis* by cDNA array. *Sci China C Life Sci* **44:** (6) 637.

Konig S, Grote J. 2002. Integrierte Funkt Genomik, Von Esmarch Str 56, DE-48149 Munster, Germany. Hydrophobic targets for MALDI mass spectrometry. *Biotechniques* **32:** (4) 912.

Kooperberg C, Fazzio TG, Delrow JJ, Tsukiyama T. 2002. Fred Hutchinson Canc Res Ctr, Div Publ Hlth Sci, 1100 Fairview Ave Nth, Seattle, Wa 98109, USA. Improved background correction for spotted DNA microarrays. *J Comput Biol* **9:** (1) 55.

Lonnstedt I, Speed T. 2002. Uppsala Univ, Dept Math, Box 480, SE-75106 Uppsala, Sweden. Replicated microarray data. *Stat Sinica* **12:** (1) 31.

McClintock TS. 2002. Univ Kentucky, Dept Physiol, 800 Rose St, Lexington, Ky 40536, USA. High-throughput expression profiling techniques. *Chem Senses* **27:** (3) 289.

Monribot-Espagne C, Boucherie H*. 2002. *Inst Biochim & Genet Cellulaires, CNRS UMR 5095, 1 rue Camille St Saens, FR-33077 Bordeaux, France. Differential gel exposure, a new methodology for the two-dimensional comparison of protein samples. *Proteomics* **2:** (3) 229.

Ramakrishnan R, Dorris D, Lublinsky A, Nguyen A, Domanus M, Prokhorova A, Gieser L, Touma E, Lockner R, Tata M *et al*. 2002. Motorola Life Sci, 4088 Commercial Ave, Northbrook, Il 60062, USA. An assessment of Motorola Codelink™ microarray performance for gene expression profiling applications. (Online art. no. E30). *Nucleic Acids Res* **30:** (7) E30.

Rickert AM, Premstaller A, Gebhardt C, Oefner PJ*. 2002. *Stanford Genome Technol Ctr, 855 Calif Ave, Palo Alto, Ca 94304, USA. Geno typing of SNPs in a polyploid genome by Pyrosequencing™. *Biotechniques* **32:** (3) 592.

Sengupta R, Tompa M. 2002. Univ Washington, Dept Comp Sci & Engn, Seig Hall, Seattle, Wa 98195, USA. Quality control in manufacturing oligo arrays: A combinatorial design approach. *J Comput Biol* **9:** (1) 1.

Warrington JA, Shah NA, Chen XY, Janis M, Liu CM, Kondapalli S, Reyes V, Savage MR, Zhang ZM *et al*. 2002. Affymetrix Inc, 3380 Cent Expressway, Santa Clara, Ca 95051, USA. New developments in high-throughput resequencing and variation detection using high density microarrays. *Hum Mutat* **19:** (4) 402.

Washburn MP, Ulaszek R, Deciu C, Schieltz DM, Yates JR. 2002. Torrey Mesa Res Inst, 3115 Merryfield Row, San Diego, Ca 92121, USA. Analysis of quantitative proteomic data generated *via* multidimensional protein identification technology. *Anal Chem* **74:** (7) 1650.

Xu CW. 2002. Cornell Univ, Mem Sloan Kettering Canc Ctr, Mol Pharmacol Prog, New York, NY 10021, USA. High-density cell microarrays for parallel functional determinations. *Genome Res* **12:** (3) 482.

### 16 Bioinformatics

Aggarwal G, Ramaswamy R*. 2002. *Jawaharlal Nehru Univ, Sch Phys Sci, IN-110067 New Delhi, India. *Ab initio* gene identification: Prokaryote genome annotation with GeneScan and GLIMMER. *J Biosci* **27:** (1 Suppl 1) 7.

Aizenberg I, Myasnikova E*, Samsonova M, Reinitz J. 2002. *Inst High Performance Comp & Databases, 118 Fontanka emb, RU-198005 St Petersburg, Russia. Temporal classification of *Drosophila* segmentation gene expression patterns by the multi-valued neural recognition method. *Math Biosci* **176:** (1) 145.

Blythe MJ, Doytchinova IA, Flower DR*. 2002. *Edward Jenner Inst Vaccine Res, Compton RG0 7NN, England. JenPep: A database of quantitative functional peptide data for immunology. *Bioinformatics* **18:** (3) 434.

Bryan J, Pollard KS, Van der Laan MJ. 2002. Univ BC, Biotechnol Lab, 333-6356 Agricultural Rd, Vancouver, Brit Columbia, Canada V6T 1Z2. Paired and unpaired comparison and clustering with gene expression data. *Stat Sinica* **12:** (1) 87.

Buchan DWA, Shepherd AJ, Lee D, Pearl FMG, Rison SCG, Thornton JM, Orengo CA*. 2002. *UCL, Dept Biochem & Mol Biol, Gower St, London WC1E 6BT, England. Gene3D: Structural assignment for whole genes and genomes using the CATH domain structure database. *Genome Res* **12:** (3) 503.

Chaudhuri P, Das S. 2002. Indian Stat Inst, Theoret Stat & Math Unit, 203 BT Rd, IN-700108 Kolkata, India. SWORDS: A statistical tool for analysing large DNA sequences. *J Biosci* **27:** (1 Suppl 1) 1.

Chen GX, Jaradat SA, Banerjee N, Tanaka TS, Ko MSH, Zhang MQ. 2002. CSH Lab, 1 Bungtown Rood, Cold Spring Harbor, NY 11724, USA. Evaluation and comparison of clustering algorithms in analyzing ES cell gene expression data. *Stat Sinica* **12:** (1) 241.

Chiaromonte F, Martinelli J. 2002. Penn State Univ, Dept Stat, University Park, Pa 16802, USA. Dimension reduction strategies for analyzing global gene expression data with a response. *Math Biosci* **176:** (1) 123.

Chilingaryan A, Gevorgyan N, Vardanyan A, Jones D, Szabo A*. 2002. *Univ Utah, Huntsman Canc Inst, 2000 Circle Hope, Salt Lake City,

Copyright © 2002 John Wiley & Sons, Ltd.

Ut 84112, USA. Multivariate approach for selecting sets of differentially expressed genes. *Math Biosci* **176:** (1) 59.
Chu TM, Weir B, Wolfinger R*. 2002. *NC State Univ, Bioinformatics Res Ctr, Raleigh, NC 27695, USA. A systematic statistical linear modeling approach to oligonucleotide array experiments. *Math Biosci* **176:** (1) 35.
Didier G, Brezellec P, Remy E, Henaut A. 2002. Lab Genome & Informatique, Tour Evry 2, 523 Pl Terrasses Agora, FR-91034 Evry, France. GeneANOVA: Gene expression analysis of variance. *Bioinformatics* **18:** (3) 490.
Ding Y. 2002. NY State Dept Hlth, Wadsworth Ctr Labs & Res, Bioinformat Lab, Albany, NY 12201, USA. Rational statistical design of antisense oligonucleotides for high throughput functional genomics and drug target validation. *Stat Sinica* **12:** (1) 273.
Dougherty ER, Barrera J, Brun M, Kim S, Cesar RM, Chen YD, Bittner M, Trent JM. 2002. Texas A&M Univ, Dept Elect Engn, 3128 TAMU, College Station, Tx 77843, USA. Inference from clustering with application to gene-expression microarrays. *J Comput Biol* **9:** (1) 105.
Dudoit S, Yang YH, Callow MJ, Speed TP. 2002. Univ Calif, Sch Publ Hlth, 140 Earl Warren Hall, Berkeley, Ca 94720, USA. Statistical methods for identifying differentially expressed genes in replicated cDNA microarray experiments. *Stat Sinica* **12:** (1) 111.
Ettinger M. 2002. Los Alamos Natl Lab, POB 1663, Los Alamos, NM 87545, USA. The complexity of comparing reaction systems. *Bioinformatics* **18:** (3) 465.
Fellenberg K, Hauser NC, Brors B, Hoheisel JD, Vingron M. 2002. German Canc Res Ctr, Dept Theoret Bioinformatics, POB 10.19.49, DE-69009 Heidelberg, Germany. Microarray data warehouse allowing for inclusion of experiment annotations in statistical analysis. *Bioinformatics* **18:** (3) 423.
Goldstein DR, Ghosh D, Conlon EM. 2002. UCLA, Dept Stat, Los Angeles, Ca, USA. Statistical issues in the clustering of gene expression data. *Stat Sinica* **12:** (1) 219.
Gu X, Van der Velden K. 2002. Iowa State Univ, Prog Bioinformat & Computat Biol, Ames, Ia 50011, USA. DIVERGE: Phylogeny-based analysis for functional-structural divergence of a protein family. *Bioinformatics* **18:** (3) 500.
Harris SE, Harris MA. 2001. Univ Missouri, Dept Oral Biol, 650 East 25th St, Kansas City, Mo 64108, USA. Gene expression profiling in osteoblast biology: Bioinformatic tools. *Mol Biol Rep* **28:** (3) 139.
Jones J, Field JK, Risk JM*. 2002. *Univ Liverpool, Mol Genet & Oncol Grp, Daulby St, Liverpool L69 3GN, England. A comparative guide to gene prediction tools for the bioinformatics amateur. *Int J Oncol* **20:** (4) 697.
Kahsay RY, Wan GL, Dongre N, Gao G, Dunbrack RL*. 2002. *Fox Chase Canc Ctr, 7701 Burholme Ave, Philadelphia, Pa 19111, USA. CASA: A server for the critical assessment of protein sequence alignment accuracy. *Bioinformatics* **18:** (3) 496.
Kaur H, Raghava GPS*. 2002. *Inst Microbial Technol, Sector 39A, Chandigarh, India. BetaTPred: Prediction of β-turns in a protein using statistical algorithms. *Bioinformatics* **18:** (3) 498.
Kerr MK, Afshari CA, Bennett L, Bushel P, Martinez J, Walker NJ, Churchill GA. 2002. Univ Washington, Dept Biostat, Box 357232, Seattle, Wa 98195, USA. Statistical analysis of a gene expression microarray experiment with replication. *Stat Sinica* **12:** (1) 203.
Kuo WP, Jenssen TK, Butte AJ, Ohno-Machado L, Kohane IS. 2002. Childrens Hosp, Informatics Program, 300 Longwood Ave, Boston, Ma 02115, USA. Analysis of matched mRNA measurements from two different microarray technologies. *Bioinformatics* **18:** (3) 405.
Lazaridis EN, Sinibaldi D, Bloom G, Mane S, Jove R. 2002. Int Agcy Res Canc, 150 Cours Albert Thomas, FR-69372 Lyon 08, France. A simple method to improve probe set estimates from oligonucleotide arrays. *Math Biosci* **176:** (1) 53.
Lazzeroni L, Owen A. 2002. Stanford Univ, Div Biostat, Redwood Bldg, Stanford, Ca 94305, USA. Plaid models for gene expression data. *Stat Sinica* **12:** (1) 61.
Lee C, Grasso C, Sharlow MF. 2002. UCLA, Dept Chem & Biochem, Los Angeles, Ca 90095, USA. Multiple sequence alignment using partial order graphs. *Bioinformatics* **18:** (3) 452.
Leplae R, Hubbard TJP. 2002. Sanger Ctr, Wellcome Trust Genome Campus, Hinxton CB10 1SA, England. MaxBench: Evaluation of sequence and structure comparison methods. *Bioinformatics* **18:** (3) 494.
Li C, Chen CC, Yang TH, Tzai TS, Huang CH, Liou JT, Su IJ, Tzeng CC, Chiu AW, Shieh B*. 2002. *Chung Shan Med Univ, Dept Biochem, 110 Chien Kuo Nth Rd Sect 1, Taichung, Taiwan. Establishment of a mini-gene expression database for bladder tumor. *J Formos Med Assoc* **101:** (2) 104.
Li KC, Yan M, Yuan SS. 2002. UCLA, Dept Stat, Math Sci Bldg, Box 951554, Los Angeles, Ca 90095, USA. A simple statistical model for depicting the cdc15-synchronized yeast cell-cycle regulated gene expression data. *Stat Sinica* **12:** (1) 141.
Li L. 2002. Fl State Univ, Dept Stat, Tallahassee, Fl 32306, USA. DNA sequencing and parametric deconvolution. *Stat Sinica* **12:** (1) 179.
Ma B, Tromp J, Li M. 2002. Univ Western Ontario, Dept Comp Sci, London, Ontario, Canada N6A 5B8. PatternHunter: Faster and more sensitive homology search. *Bioinformatics* **18:** (3) 440.
McLachlan GJ, Bean RW, Peel D. 2002. Univ Queensland, Dept Math, Brisbane, Qld 4072, Australia. A mixture model-based approach to the clustering of microarray expression data. *Bioinformatics* **18:** (3) 413.
Mills AP. 2002. Univ Calif, Dept Phys, Riverside, Ca 92521, USA. Gene expression profiling diagnosis through DNA molecular computation. *Trends Biotechnol* **20:** (4) 137.
Nandi T, B-Rao C, Ramachandran S*. 2002. *Ctr Biochem Technol, Funct Genomics Unit, Mall Rd, IN-110007 Delhi, India. Comparative genomics using data mining tools. *J Biosci* **27:** (1 Suppl 1) 15.
Ness SR, Terpstra W, Krzywinski M, Marra MA, Jones SJM*. 2002. *BC Canc Agcy, Genome Sequence Ctr, 600 West 10th Ave, Vancouver, Brit Columbia, Canada V5Z 4E6. Assembly of fingerprint contigs: Parallelized FPC. *Bioinformatics* **18:** (3) 484.
Pollard KS, Van der Laan MJ*. 2002. *Univ Calif, Dept Biostat, Warren Hall 7360, Berkeley, Ca 94720, USA. Statistical inference for simultaneous clustering of gene expression data. *Math Biosci* **176:** (1) 99.
Rouillard JM, Herbert CJ, Zuker M. 2002. Univ Michigan, Dept Chem Engn, Ann Arbor, Mi 48109, USA. OligoArray: Genome-scale oligonucleotide design for microarrays. *Bioinformatics* **18:** (3) 486.
Sabatti C, Karsten SL, Geschwind DH. 2002. UCLA, Dept Human Genet & Stat, 695 Charles Young Dr Sth, Los Angeles, Ca 90095, USA. Thresholding rules for recovering a sparse signal from microarray experiments. *Math Biosci* **176:** (1) 17.
Schachter V. 2002. Hybrigen, 3-5 Impasse Reille, FR-75014 Paris, France. Bioinformatics of large-scale protein interaction networks. *Biotechniques* (Suppl) 16.
Schmidt HA, Strimmer K, Vingron M, Von Haeseler A*. 2002. *Max Planck Inst Evolutionare Anthropol, Inselstr 22, DE-04103 Leipzig, Germany. TREE-PUZZLE: Maximum likelihood phylogenetic analysis using quartets and parallel computing. *Bioinformatics* **18:** (3) 502.
Shedden K. 2002. Univ Michigan, Dept Stat, 105 Sth State St, Ann Arbor, Mi 48109, USA. Modeling the influence of disease processes on gene expression. *Stat Sinica* **12:** (1) 263.
Szabo A, Boucher K, Carroll WL, Klebanov LB, Tsodikov AD, Yakovlev AY. 2002. Univ Utah, Huntsman Canc Inst, 2000 Circle Hope, Salt Lake City, Ut 84112, USA. Variable selection and pattern recognition with gene expression data generated by the microarray technology. *Math Biosci* **176:** (1) 71.
Tammi MT, Arner E, Britton T, Andersson B*. 2002. *Uppsala Univ, Dept Genet & Pathol, Uppsala, Sweden. Separation of nearly identical repeats in shotgun assemblies using defined nucleotide positions, DNPs. *Bioinformatics* **18:** (3) 379.
Tesler G. 2002. Univ Calif San Diego, Dept Comp Sci & Engn, La Jolla, Ca 92093, USA. GRIMM: Genome rearrangements web server. *Bioinformatics* **18:** (3) 492.
Therneau T, Tschumper RC, Jelinek D. 2002. Mayo Clin & Mayo Fdn, Dept Hlth Sci Res, 200 1st St SW, Rochester, Mn 55905, USA. Sharpening spots: Correcting for bleedover in cDNA array images. *Math Biosci* **176:** (1) 1.
Tibshirani R, Hastie T, Narasimhan B, Eisen M, Sherlock G, Brown P, Botstein D. 2002. Stanford Univ, Dept Hlth Res & Policy & Stat, Redwood Bldg, Stanford, Ca 94305, USA. Exploratory screening of genes and clusters from microarray experiments. *Stat Sinica* **12:** (1) 47.
Vincens P, Badel-Chagnon A, Andre C, Hazout S. 2002. Ecole Normale Super, Dept Biol, 46 rue Ulm, FR-75230 Paris 05, France. D-ASSIRC: Distributed program for finding sequence similarities in genomes. *Bioinformatics* **18:** (3) 446.
Wang PL, Ding F, Chiang HY, Thompson RC, Watson SJ, Meng F. 2002. Univ Michigan, Dept Psychiat, 205 Zina Pitcher Pl, Ann Arbor, Mi 48109, USA. ProbeMatchDB: A web database for finding equivalent probes across microarray platforms and species. *Bioinformatics* **18:** (3) 488.

Copyright © 2002 John Wiley & Sons, Ltd. 
